# Health Capital

**Published:** 1880-01

**Authors:** 


### HEALTH CAPITAL.

Facts become impressive when stated so as at once to appeal to the judgment. No plan is so forcible as that of drawing comparisons. When we say that health is destroyed by dissipation, we state a fact, but it is trite and hackneyed from its constant repetition, and, therefore, fails to produce the effect it should. The same truth presented in another form, to a person of quick perception, becomes startling; as when we say that every living creature born into the world in sound health, possesses this health, as his capital, which, if properly employed, returns a certain income in health and happiness, without impairing the principal; that every excess, every dissipation indulged in, is at the expense of this capital, which it is impossible ever again to restore; the statement is within the comprehension of every one. One of the characters in "Gil Blas" is a young Spaniard whose inheritance was a large sum of money. He was educated to no business, and desired none, his sole occupation being to enjoy life. Instead of investing his money where it would bring him a regular income without encroaching on the principal, he divided it into as many parts as the number of years he was likely to live, which gave him a generous sum each year, and he then gave himself up to what he considered pleasure—an indolent and dissipated life.

This is really what thousands of young men are doing to-day. They have been left with ample means, but with no regular occupations in life, and no incentives to industry.

They pursue the delusive phantom—pleasure—until health and fortune are lost, and at the age when a temperate man is just prepared to enter upon a life of usefulness, with the prospect of health, happiness, and a long life before him, these wrecks drop into their graves unwept and unhonored. There are thousands of persons who inherit fortunes and make good use of them, and there are thousands of good men who, for the want of means, struggle through life in honest poverty, so that no rule would apply to all cases ; but if every young man should be bred to habits of industry and sobriety, and have a regular business, of which he was master, there would be fewer wrecks, fewer crimes, and more general prosperity.

